# Smoothelin-Like Protein 1 Regulates Development and Metabolic Transformation of Skeletal Muscle in Hyperthyroidism

**DOI:** 10.3389/fendo.2021.751488

**Published:** 2021-10-05

**Authors:** Evelin Major, Ferenc Győry, Dániel Horváth, Ilka Keller, István Tamás, Karen Uray, Péter Fülöp, Beáta Lontay

**Affiliations:** ^1^ Department of Medical Chemistry, Faculty of Medicine, University of Debrecen, Debrecen, Hungary; ^2^ Department of Surgery, Faculty of Medicine, University of Debrecen, Debrecen, Hungary; ^3^ Department of Internal Medicine, Division of Metabolism, Faculty of Medicine, University of Debrecen, Debrecen, Hungary

**Keywords:** insulin sensitivity, skeletal muscle, hyperthyroidism, insulin signaling, insulin receptor substrate 1, phosphorylation, glucose metabolism

## Abstract

Hyperthyroidism triggers a glycolytic shift in skeletal muscle (SKM) by altering the expression of metabolic proteins, which is often accompanied by peripheral insulin resistance. Our previous results show that smoothelin-like protein 1 (SMTNL1), a transcriptional co-regulator, promotes insulin sensitivity in SKM. Our aim was to elucidate the role of SMTNL1 in SKM under physiological and pathological 3,3′,5-Triiodo-L-thyronine (T3) concentrations. Human hyper- and euthyroid SKM biopsies were used for microarray analysis and proteome profiler arrays. Expression of genes related to energy production, nucleic acid- and lipid metabolism was changed significantly in hyperthyroid samples. The phosphorylation levels and activity of AMPKα2 and JNK were increased by 15% and 23%, respectively, in the hyperthyroid samples compared to control. Moreover, SMTNL1 expression showed a 6-fold decrease in the hyperthyroid samples and in T3-treated C2C12 cells. Physiological and supraphysiological concentrations of T3 were applied on differentiated C2C12 cells upon SMTNL1 overexpression to assess the activity and expression level of the elements of thyroid hormone signaling, insulin signaling and glucose metabolism. Our results demonstrate that SMTNL1 selectively regulated TRα expression. Overexpression of SMTNL1 induced insulin sensitivity through the inhibition of JNK activity by 40% and hampered the non-genomic effects of T3 by decreasing the activity of ERK1/2 through PKCδ. SMTNL1 overexpression reduced IRS1 Ser307 and Ser612 phosphorylation by 52% and 53%, respectively, in hyperthyroid model to restore the normal responsiveness of glucose transport to insulin. SMTNL1 regulated glucose phosphorylation and balances glycolysis and glycogen synthesis *via* the downregulation of hexokinase II by 1.3-fold. Additionally, mitochondrial respiration and glycolysis were measured by SeaHorse analysis to determine cellular metabolic function/phenotype of our model system in real-time. T3 overload strongly increased the rate of acidification and a shift to glycolysis, while SMTNL1 overexpression antagonizes the T3 effects. These lines of evidence suggest that SMTNL1 potentially prevents hyperthyroidism-induced changes in SKM, and it holds great promise as a novel therapeutic target in insulin resistance.

## Introduction

Hyperthyroidism is a pathological disorder in which excess thyroid hormone (TH) is produced and secreted by the thyroid gland (1). Thyroid hormones are key regulators of metabolism and development and have diverse effects. Thyroxine (T4) is the main product of thyroid secretion. Thyroxine is deiodinated in peripheral tissues to produce 3,3′,5-Triiodo-L-thyronine (T3), the biologically active TH (2). The biological effects of THs are mediated through the interaction of T3 with high-affinity thyroid receptors (TRs). TRs are ligand-dependent nuclear transcription factors that interact with thyroid-responsive elements (TREs) located in the promoter of T3-responsive genes (3). Five different subtypes of TRs (α1, α2, β1, β2, and β3) are encoded by two genes, TRα (*NR1A1*) and TRβ (*NR1A2*). All subtypes except TRα2 bind T3 (4, 5).

THs have profound effects on skeletal muscle (SKM), affecting contractile function, metabolic properties, myogenesis, and muscle regeneration *via* altered gene expression (6, 7). THs cross the plasma membrane by facilitated diffusion that is primarily mediated by monocarboxylate transporters 10 and 8 (MCT10 and 8) in SKM (8, 9). Intracellular TH levels are also modulated by iodothyronine deiodinase type 2 (DIO2), which converts T4 to T3 to increase intracellular T3 availability and effects (10). T3 regulates gene expression in SKM by interacting with TRα and TRβ at specific promoter regions (11, 12). In addition, THs elicit short-term effects in SKM by regulating membrane transporter activity (13), phospholipase C (14), and the kinase activity of p38 and AMP-activated protein kinase (AMPK), which are important in mitochondrial biogenesis (15).

SKM represents 40% of the human body mass and is one of the most important tissues regulating energy expenditure and lipid and glucose homeostasis (16). Changes in the energetic profile of SKM significantly affect systemic physiology (17). Insulin-mediated glucose uptake by SKM is regulated through the phosphatidylinositol 3-kinase (PI3K) and protein kinase B (PKB/Akt) pathways and the heterologous MAPKs and AMPK pathways. Insulin binds to the insulin receptor (IR) triggering an intracellular signaling cascade that includes the phosphorylation and binding of insulin receptor substrate 1 (IRS1), PI3K, and Akt. IRS1 provides an interacting surface for both IR and PI3K; thus, the regulation of IRS1 is crucial (18). As the target of insulin-dependent cascades, glucose disposal is increased by the translocation of glucose transport molecule 4 (GLUT4) from the intracellular vesicles to the surface and by the regulation of glucose transport, glucose phosphorylation, glycogen synthesis, glycolysis, and glucose oxidation (19). T3 promotes an elevation in basal and some extent insulin-mediated glucose uptake primarily increasing GLUT4 protein expression and insulin-independent translocation of GLUT4 (20). Interestingly, T3 also exerts acute, non-genomic action by activating GLUT4 already present on the plasmamembrane (21).

One potential regulator of insulin resistance in SKM is the smoothelin-like protein 1 (SMTNL1). SMTNL1 was identified as an early target of protein kinase A and G (PKA/PKG) in gastrointestinal smooth muscle (22) and is expressed in skeletal muscle and steroid hormone-sensitive tissues. SMTNL1 translocates to the nucleus after phosphorylation at Ser301 by PKA/PKG. In the nucleus, SMTNL1 functions as a transcriptional regulator of progesterone receptors, leading to subsequent gene regulation of numerous metabolic enzymes, cytoskeletal elements, steroid receptors, and cytokines (23). We previously showed that SMTNL1 regulates insulin signaling by promoting the gene expression of GLUT4 and IRS1 in murine skeletal muscle. Moreover, SKM fiber type shifts from oxidative to glycolytic phenotype in *SMTNL1* KO mice; consequently, *SMTNL1^-^* ^/-^ mice are metabolically less efficient and exhibit impaired glucose tolerance (24).

SMTNL1 is the major metabolic regulator in SKM and SMTNL1 KO mice mimic the pathophysiological processes of hyperthyroidism (24). Thus, our aim was to determine the role of SMTNL1 in SKM under physiological and pathological TH concentrations. We found that THs play diverse roles in the regulation of SKM development and homeostasis through the downregulation of SMTNL1 expression. SMTNL1 overexpression prevented the metabolic shift of SKM during insulin resistance induced by supraphysiological TH levels.

## Materials and Methods

### Chemicals

All chemicals were obtained from Sigma-Aldrich (St. Louis, MO, USA) unless otherwise indicated.

### Antibodies

See **Table 1** in the **Supplementary Materials and Methods** for a list of antibodies used in Western blot analyses. See **Figure S7** for evidence that β-actin is an applicable loading control in our model.

### Cell Culture Maintenance and Transfection

C2C12 mouse myoblast cells (*ECACC 91031101*) were grown in Dulbecco’s Modified Eagle’s Medium (DMEM) supplemented with 1000 mg/L glucose, 2 mM L-glutamine (Lonza), 10% (v/v) fetal bovine serum (FBS; Sigma-Aldrich; St. Louis, MO, USA; Cat. No. F9665, batch number: BCBW5069), and phenol red at 37°C and 5% CO_2_. Transient transfection was conducted using GeneJuice reagent with pM13-NT-FT-SMTNL1 (NT-FT: N-terminal Flag tagged SMTNL1) as described in **Supplementary Materials and Methods**. Myoblasts at passage number 6-9 were seeded into collagen-coated tissue culture plates or dishes (VWR International; West Chester, PA, USA) and incubated overnight (O/N). Transfection with empty vector (MOCK) acted as the control in all experiments.

### Differentiation and Treatment of C2C12 Cells

The day after transfection, myogenic differentiation was induced at 90% confluency. Cells were washed with 1x Phosphate buffered saline (PBS) and the growth medium was replaced by phenol red-free DMEM supplemented with 1000 mg/L glucose, 2 mM L-glutamine and 2% (v/v) horse serum (HS) (differentiation medium). 10 nM T3 dissolved in 1 M HCl : EtOH (1:4) was added from the first day (**Figure 4**) or from the beginning of the fourth day (**Figures 5**–**8**) of differentiation. The non-treated control cells were treated with 1 M HCl : EtOH and are called ‘vehicle’ in the figures. Differentiation medium supplemented with either T3 or vehicle was changed daily for 6 days (**Figure 4**) and for 72 hours (**Figures 5**–**8**).

### SeaHorse Analysis

Myoblasts were transfected with empty vector or NT-FT-SMTNL1 and were seeded at a density of 10 000 cells per well in collagen-coated XF96 microplates (Agilent Technologies; Santa Clara, CA, USA). Following a 72-hour T3 treatment of cells, 180 µl of assay media (Agilent Technologies; Santa Clara, CA, USA) containing 1000 mg/L glucose was added. Myoblasts were incubated for 1 hour in a non-CO_2_ incubator along with the previously hydrated sensor cartridge (Agilent Technologies; Santa Clara, CA, USA) loaded with the following inhibitors: 50 µM etomoxir (carnitine palmitoyltransferase-1 inhibitor); 2 µM oligomycin (ATP synthase inhibitor); 4 µM FCCP (carbonyl cyanide-p-trifluoromethoxyphenylhydrazone; mitochondrial oxidative phosphorylation uncoupler), or 10 µM antimycin (mitochondrial electron transport chain complex III inhibitor) + 100 mM 2-deoxy-D-glucose (glycolysis inhibitor). Five measurement points were taken for the baseline and after each injection. Finally, cells were solubilized in 1 N NaOH and total protein concentration was measured using a Bicinchoninic acid (BCA) assay kit (Thermo Fisher Scientific; Waltham, MA, USA).

### Cell Lysis

On the last day of differentiation combined with SMTNL1 overexpression and/or hormone treatment, myotubes were washed with 1x PBS and were harvested in ice-cold RIPA buffer [25 mM Tris-HCl (pH 7.6), 150 mM NaCl, 1% sodium deoxycholate, 0.1% SDS, 1% Triton-X 100, 1mM PMSF, 10x PIC, 50x PPIC, 1 µM MC-LR] on ice. Cell suspensions were sonicated for 30 seconds with a 10% pulse using a Branson Sonifier sonicator and were centrifuged for 10 minutes at 16 000 x *g*, 4°C. Then supernatants were transferred to fresh tubes. Total protein concentrations were measured by Bicinchoninic acid (BCA) assay kit (Thermo Fisher Scientific; Waltham, MA, USA).

### Western Blot Analysis

After harvesting and lysing cells, whole-cell lysates were boiled in 4x sodium dodecyl sulphate (SDS) sample buffer (Bio-Rad Laboratories; Hercules, CA, USA) for 5 minutes. Western blot analyses were conducted as described previously (25) with the following modifications: 30 µg of protein was loaded onto 4-20% precast Criterion gels (Bio-Rad Laboratories; Hercules, CA, USA) and separated at 200 V. After a 75-minute transfer at 100 V, nitrocellulose membranes were blocked in 5% (w/v) bovine serum albumin (BSA)/Tris-buffered saline (TBS) containing 0.1% (v/v) Tween 20 for 1.5 hours. The blots were incubated with primary antibodies at 4°C, O/N and secondary antibodies at room temperature (RT) for 1.5 hours, diluted in the blocking solution. In phospho-blots, membranes were stripped or HRP-inactivated as described in **Supplementary Materials and Methods** and were re-probed with non-phospho antibodies.

### Human Euthyroid and Hyperthyroid Skeletal Muscle (SKM) Biopsies

Sternohyoid muscle biopsies from euthyroid (eu) and hyperthyroid (hyper) donors were frozen in liquid N_2_ at the time of harvest and were stored at -70°C. Donors were selected based on their gender and disease. All biopsies were derived from female patients with struma nodosa (euthyroid) or with Basedow or struma nodosa colloides (hyperthyroid). We strove to select female patients not older than 60 years (**Figure S1**). Samples were homogenized in modified RIPA buffer [25 mM Tris (pH 7.6), 150 mM NaCl, 0.1% SDS, 1% Triton X-100, 1 mM EDTA, 1 mM DTT, 1 mM PMSF, 10x PIC, 50x PPIC, 1 µM MC-LR] on ice using a glass Potter-Elvehjem tissue grinder. After centrifugation at 16 000 x *g*, 4°C, supernatants were transferred to fresh tubes and were subjected to Western blot analysis. Statistical analysis was performed using data from 73 patients, as shown in **Supplementary Figures**.

### Total RNA Isolation for Microarray Analysis of Human SKM Biopsies

An RNeasy Fibrous Tissue kit (cat. no. 74704, Qiagen; Hilden, Germany) was used according to the manufacturer’s instructions. Euthyroid and hyperthyroid SKM samples (3 of each) were homogenized in a guanidine-isothiocyanate buffer using the TissueRuptor (Qiagen; Hilden, Germany) and were treated with proteinase K at 55°C for 10 minutes. Cell debris was removed by centrifugation at 10 000 x *g* for 3 minutes and supernatants were transferred to fresh tubes. Then 96-100% ethanol was added to the cleared lysates and was mixed thoroughly. After RNA was bound to the RNeasy silica membrane, traces of DNA were eliminated by DNase I treatment on the RNeasy column at 30°C for 15 minutes. DNase I and any possible contaminants were washed away, and RNA was eluted in RNase-free water. Total RNA concentration and purity were determined using a NanoDrop ND-1000 spectrophotometer (Thermo Fisher Scientific; Waltham, MA, USA). The microarray analysis was performed using an Affymetrix Human Gene 1.0 ST Array as previously described (26). All of the raw DNA chip data is available in the Gene Expression Omnibus (GEO; http://www.ncbi.nlm.nih.gov/geo/) under the following accession number: GSE178996.

### Proteome Profiler Human Phospho-Kinase and Phospho-MAPK Arrays

Human Phospho-Kinase (cat. no. ARY003B) and Phospho-MAPK (cat. no. ARY002B) array kits (R&D Systems; Minneapolis, MN, USA) were used according to the manufacturer’s instructions. Briefly, human euthyroid and hyperthyroid SKM biopsies were homogenized in Lysis Buffer 6 supplemented with protease and phosphatase inhibitors. For the Phospho-Kinase Array, samples were diluted in Array Buffer 1 and were incubated with the membranes at 4°C O/N. On the next day, membranes were incubated in a cocktail of biotinylated detection antibodies at RT for 2 hours. For the Phospho-MAPK Array, samples were diluted in Array Buffer 1 and were incubated with Detection Antibody Cocktail at RT for 1 hour. Then the prepared sample/antibody mixtures were added to the membranes which were incubated at 4°C O/N. Membranes were then incubated in diluted Streptavidin-HRP and the signal was detected by ECL with the provided Chemi-Reagent Mix in both arrays.

### Statistical Analysis

Immunoblots were analyzed using ImageJ. Bar charts were created and statistical analyses were performed using GraphPad Prism8. Phosphorylated proteins were normalized to non-phosphorylated protein expression, while non-phosphorylated protein expression was normalized to the loading control. Normalized data were checked for normality and were analyzed using unpaired two-tailed t-test for the comparison of two groups, and One-way ANOVA or Two-way ANOVA for the comparison of four or more groups followed by their corresponding parametric or non-parametric *post hoc* test. All data are presented as mean +/- SEM, where n is the number of independently performed experiments. Outliers were identified using the Thompson Tau-test and Grubbs’s test. Differences were considered statistically significant at p <0.05.

## Results

### Microarray Analysis of Euthyroid and Hyperthyroid Human Skeletal Muscle Tissues and a Gene Ontology Analysis

**Figure 1:**
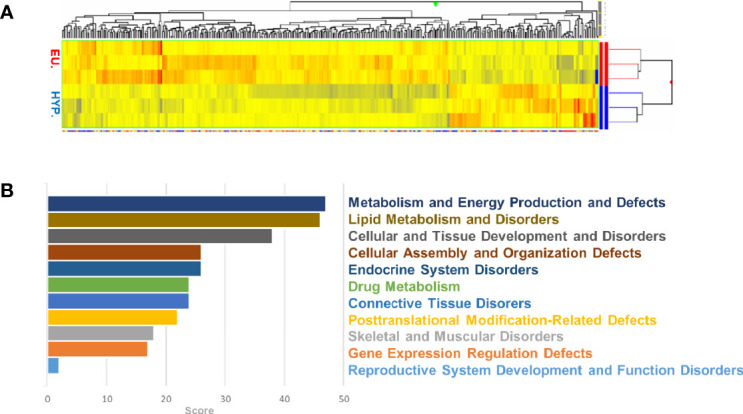
Microarray analysis of human euthyroid and hyperthyroid skeletal muscle biopsies. Euthyroid and hyperthyroid SKM samples were analyzed by microarray. The color code for the signal strength is shown in which induced genes are indicated by red and repressed genes are indicated by blue **(A)**. Ontology of the related genes and their classification by GO terms **(B)**.

A tissue bank with 73 samples of sternohyoid muscle from 44 euthyroid and 29 hyperthyroid patients undergoing thyroid surgery was established. Using patient data, statistical analysis was performed based on the classification of donors (**Figure S1**). Over 39,000 transcripts were examined, encompassing the entire expressed human genome. Comparing the euthyroid and hyperthyroid samples, 393 differentially regulated genes were identified. **Figure 1** shows a list of genes that were up- or downregulated (p<0.05; *n* = 3) in the hyperthyroid samples compared with gene expression in the euthyroid samples. Hierarchical cluster analysis was performed on all differentially expressed genes using average linkage with Pearson’s dissimilarity and the numbers of induced or repressed genes are presented in a heat map (**Figure 1A**). Hyperthyroidism influenced molecular cellular functions and their related diseases, including disorders of metabolism and energy production, lipid metabolism, cellular and tissue development, endocrine system, skeletal and muscular function, and gene expression regulation (**Figure 1B**). Bioinformatics analysis identified differentially regulated canonical pathways; the most profound changes were found in mTOR, p70S6K, AMPK, PI3K/Akt and MAPK signaling pathways (**Figure S2**). Taken together, the signaling pathways identified by microarray analysis showed altered expression of genes involved in metabolism, development, and cytoskeletal function in SKM upon the clinical manifestations of hyperthyroidism.

### Hyperthyroidism Affects Key Metabolic Pathways in Human Skeletal Muscle

**Figure 2:**
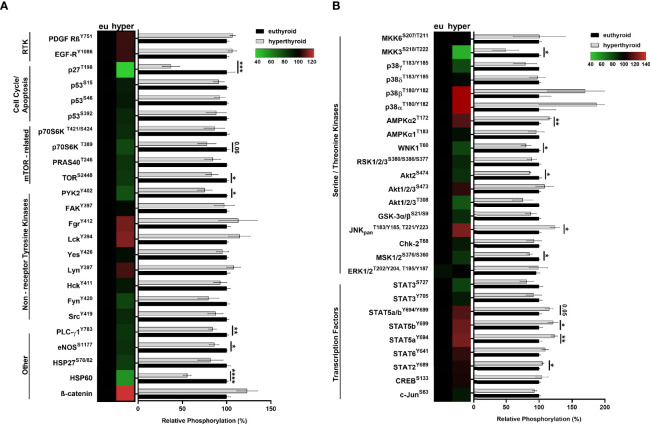
Analysis of human euthyroid and hyperthyroid skeletal muscle biopsies with proteome profiler phospho-kinase and phospho-MAPK arrays. **(A, B)** Euthyroid and hyperthyroid SKM samples were analyzed by human Phospho-Kinase array and by Human Phospho-MAPK Array. Results were visualized by a combination of heat maps and graphs. Bar charts show the phosphorylation levels of various proteins in hyperthyroid samples relative to their corresponding euthyroid pairs. In case of the heat maps, increased phosphorylation levels are indicated by red and decreased phosphorylation levels are indicated by green. Values represent n=2-3, mean +/- SEM. Groups were compared using unpaired two-tailed t-tests, p < 0.05 (*), p < 0.01 (**), p < 0.001 (***), and p < 0.0001 (****).

To investigate hyperthyroidism-induced changes in human SKM, we analyzed the phosphorylation profiles of an array of kinases. The comparison of hyperthyroid and euthyroid samples were visualized using heat maps and graphs (**Figures 2A, B**). Phosphorylation levels of transcription factors, including STAT2^Y689^, STAT5a^Y694^, STAT5b^Y699^ were markedly increased in hyperthyroidism (**Figure 2B**). Among Ser/Thr kinases, phosphorylation of MSK1/2^S376/S360^, Akt2^S474^, WNK1^T60^ and MKK3^S218/T222^ was significantly reduced and phosphorylation of ERK1/2^T202/Y204,T185/Y187^, AMPKα2^T172^ and JNK_pan_^T183/Y185,T221/Y223^ was strongly elevated in hyperthyroid samples compared with phosphorylation in euthyroid samples (**Figure 2B**). Interestingly, phosphorylation of mTOR^S2448^, p70S6K^T421/S424^, PLC-γ1^Y783^, eNOS^S1177^, and HSP60 was considerably diminished in the hyperthyroid samples compared with phosphorylation in the euthyroid samples (**Figure 2A**). Regarding cell cycle and apoptosis, hyperthyroidism decreased the phosphorylation of p27^T198^ only (**Figure 2A**). These results suggest that hyperthyroidism has a huge impact on key metabolic pathways through posttranslational regulation of their members in SKM. We also further validated our results on differentiated “hyperthyroid” C2C12 cell model by semi-quantitative Western blot analysis (**Figure 6E** and **Figures 7A–C**).

### SMTNL1 Expression Is Changed in Skeletal Muscle During Hyperthyroidism

**Figure 3:**
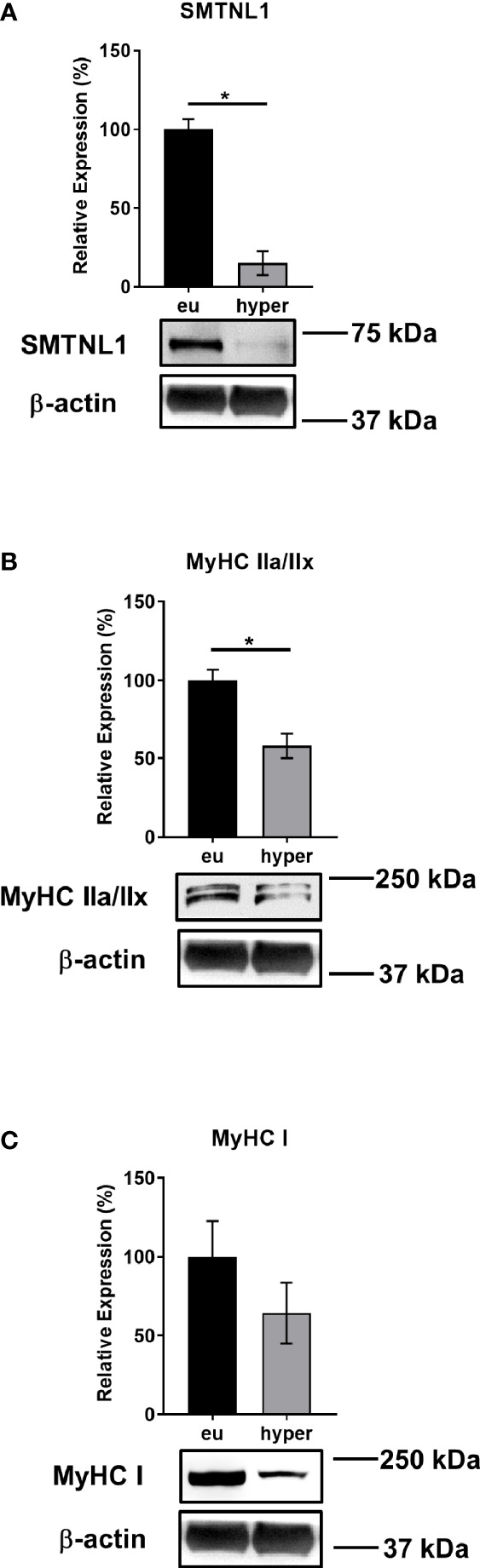
SMTNL1, MyHC IIa/IIx, and MyHC I Expression during Hyperthyroidism. Proteins from skeletal muscle biopsies were separated by SDS-PAGE followed by Western blot analysis using anti-SMTNL1 **(A)**, anti-MyHC IIa/IIx **(B),** and anti-MyHC I **(C)** antibodies. Values represent n=3, mean +/- SEM. Bar charts display the expression levels of SMTNL1, MyHC IIa/IIx, and MyHC I in hyperthyroid samples normalized to their euthyroid counterparts. Groups were compared using unpaired two-tailed t-tests, p < 0.05 (*).

To investigate the role of SMTNL1 in hyperthyroidism, human euthyroid and hyperthyroid SKM samples were analyzed by Western blot. SMTNL1 expression decreased 6-fold in the hyperthyroid samples (**Figure 3A**), indicating that SMTNL1 may play a role in the pathophysiology of hyperthyroidism in SKM. This change was accompanied by a significant decrease in the expression of type IIa fiber marker MyHC IIa (**Figure 3B**) and a considerable decrease in type I fiber marker MyHC I expression (**Figure 3C**), suggesting an SKM isotype switch and metabolic shift from an oxidative to a glycolytic fiber type induced by hyperthyroidism.

### Effects of Supraphysiological T3 Exposure on C2C12 Differentiation

**Figure 4:**
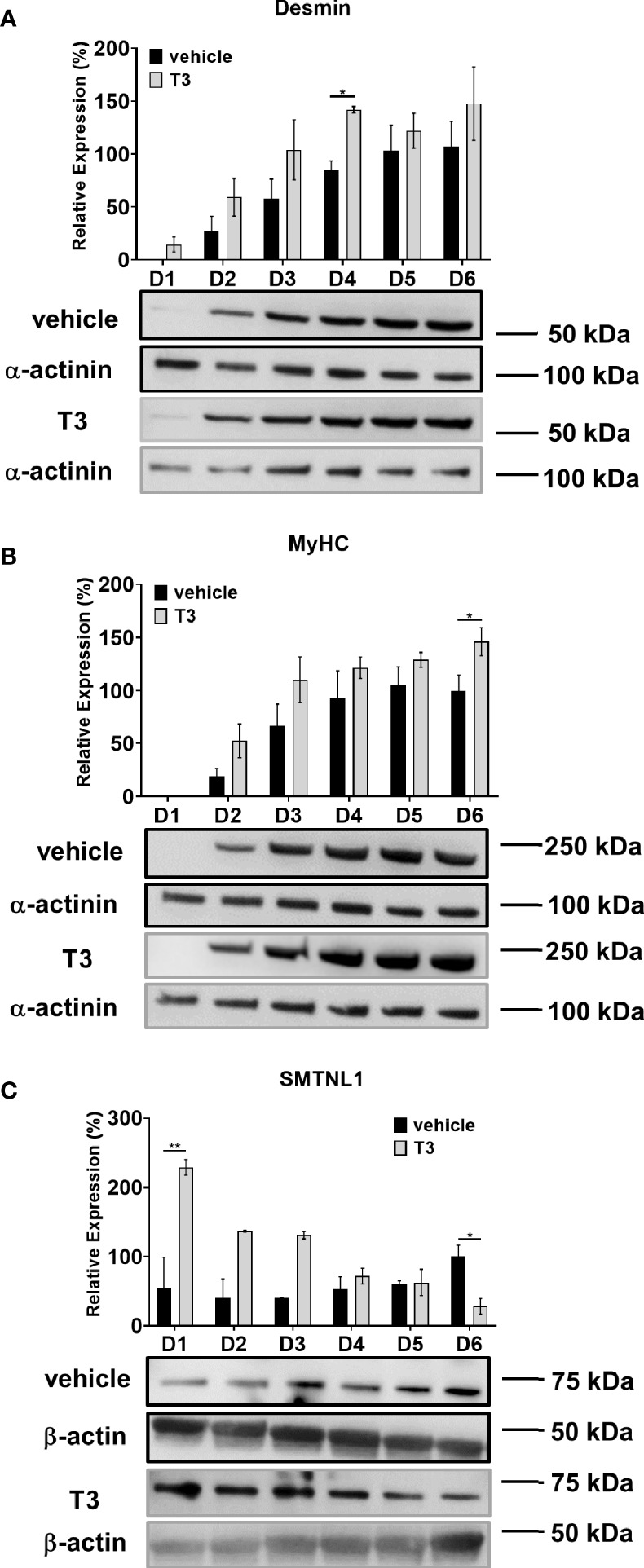
Effect of T3 treatment on SMTNL1 expression during C2C12 myogenesis. **(A–C)** Myoblasts were seeded on a collagen-coated plate and differentiation was induced in the presence of 2% HS and supraphysiological (10 nM) T3. Equal amounts of 1 M HCl : EtOH solution was added to the non-treated control, indicated as ‘vehicle’ which contains physiological (approximately 1 nM) T3. The differentiation medium was changed daily for 6 days. Myotubes were harvested and lysates were analyzed by Western blot using anti-desmin **(A)**, anti-MyHC **(B),** and anti-SMTNL1 **(C)** antibodies. Values represent n=3-4, mean +/- SEM. Data were normalized to the D6 vehicle control. Groups were compared using Two-way ANOVA and Tukey’s *post hoc* test, p < 0.05 (*), p < 0.01 (**).

The C2C12 mouse myoblast cell line was used to validate the results from the human SKM biopsies and explore the molecular mechanisms behind the effects of SMTNL in hyperthyroidism. C2C12 mouse myoblast cells are widely used to study skeletal muscle metabolism *in vitro* (27). In our model system, myoblasts were differentiated for 6 days in the presence of physiological or supraphysiological T3 (10 nM) (28, 29). Differentiation was confirmed by Western blot analysis of early (desmin) and late (MyHC) myogenic markers. Neither physiological nor supraphysiological T3 condition altered the expression of myogenic markers after Day 4. Desmin expression was significantly upregulated by 57% on Day 4, and MyHC expression was significantly upregulated by 46% on Day 6 of T3 treatment (**Figures 4A, B**). Thus, T3 promotes the myogenesis of C2C12 myoblasts into multinucleated, fiber-shaped myotubes. We also examined the effects of differentiation and T3 treatment on endogenous SMTNL1 expression. SMTNL1 expression was increased during regular differentiation; however, it was not significant. Upon T3 treatment, SMTNL1 levels were significantly increased on Day 1 but then progressively decreased 4-fold by the end of differentiation compared to the vehicle (**Figure 4C**). SMTNL1 was transiently overexpressed in C2C12 myoblasts, which were differentiated for 6 days. On the beginning of Day 4, differentiating myoblasts were treated with 10 nM of T3 for 72 hours. SMTNL1 overexpression was confirmed by Western blot analysis (**Figure S3A**). Based on these data, SMTNL1 levels were inversely altered upon T3-treatment suggesting the regulatory effect of T3 on SMTNL1 expression. We examined the effects of the previously described treatments on the viability of C2C12 cells using the alamarBlue assay. Neither SMTNL1 overexpression nor T3 treatment affected the viability of myotubes (**Figure S3B**). These results are in agreement with our Proteome Profiler data showing that hyperthyroidism does not induce apoptosis in SKM.

### T3 Treatment and SMTNL1 Overexpression Influence the Expression of Regulatory Proteins Involved in T3 Signaling/Action in C2C12 Myotubes

**Figure 5:**
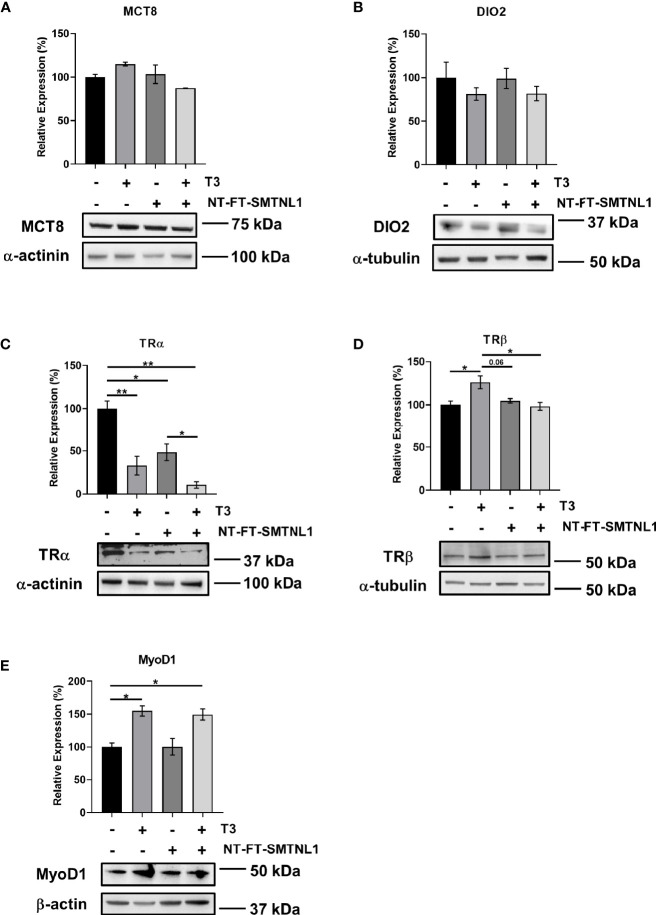
T3 treatment and SMTNL1 overexpression influence the expression of regulatory proteins involved in T3 signaling/action. **(A–C)** Myoblasts were transfected with either empty vector or NT-FT-SMTNL1 and were differentiated with a simultaneous 72-hour T3 treatment starting from Day 4. Proteins from whole-cell lysates were analyzed by Western blot using anti-MCT8 **(A)**, anti-DIO2 **(B)**, anti-TRα **(C),** anti-TRβ **(D),** and anti-MyoD1 **(E)**. Values represent n=3-5, mean +/- SEM. Data were normalized to the empty vector-transfected control. Groups were compared using One-way ANOVA and Tukey’s post hoc test, p < 0.05 (*), p < 0.01 (**).

The effects of THs are primarily determined by the presence of TH transporters in the plasma membrane, the expression of TH receptors, and intracellular hormone availability, which depend on the activity of iodothyronine deiodinases. To elucidate the effects of SMTNL1 on MCT8, DIO2, TRα, and TRβ expression upon T3 treatment, we carried out Western blot analyses on NT-FT-SMTNL1-transfected and/or T3-treated myotubes. MCT8 and DIO2 expression did not change significantly (**Figures 5A, B**). However, TRα expression was dramatically reduced by 67% and 52% in response to T3 treatment and SMTNL1 overexpression, respectively. Initially, our hypothesis was that SMTNL1 can antagonize the effects of T3. Remarkably, the combined treatment with T3 and SMTNL1 caused a further decrease in TRα expression by 37% compared to SMTNL1 overexpression but it was not significant compared to T3 treatment (**Figure 5C**). These data are in line with a previous study demonstrating that THs lower TRα levels *via* proteasomal degradation in SKM (30). In contrast, T3 treatment led to a significant increase in TRβ expression (**Figure 5D**). SMTNL1 overexpression did not significantly change TRβ protein expression (**Figure 5D**) or boost the effect of T3 on TRα expression (**Figure 5C**). Thus, SMTNL1 does not appear to antagonize the effects of T3 at TRs. Furthermore, SMTNL1 does not directly interact with TRα or TRβ, based on the immunoprecipitation of NT-FT-SMTNL1 (**Figure S4A**). Treatment of NT-FT-SMTNL1-transfected myotubes with a membrane-permeable cAMP analog, 8BrcAMP, significantly reduced TRα expression by 42% compared to SMTNL1 overexpression alone (**Figure S4B**), suggesting that SMTNL1 may regulate TRα expression at the transcriptional level. MyoD1, one of the master proteins controlling myogenesis, is positively regulated by T3 (31, 32) and our data are in agreement with this notion; T3 treatment alone or in combination with SMTNL1 overexpression induced a 55% and a 49% increase in MyoD1 expression in myotubes, respectively (**Figure 5E**). These data indicate that T3 has a positive effect on C2C12 myogenesis through the regulation of MyoD1.

### SMTNL1 Overexpression Decreases Ser Phosphorylation of IRS1 in the Presence of Excess T3 in C2C12 Myotubes

**Figure 6:**
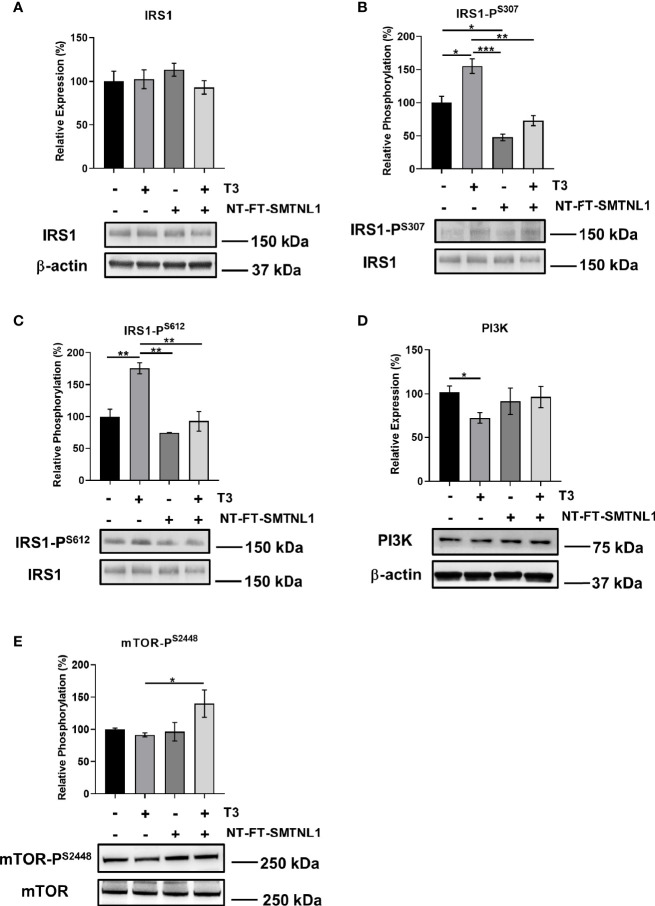
T3 overload and SMTNL1 overexpression affect the phosphorylation of key members of insulin signaling. **(A–E)** Empty vector or NT-FT-SMTNL1-transfected myoblasts were differentiated with a simultaneous 72-hour T3 treatment starting from Day 4. Proteins from whole-cell lysates were analyzed by Western blot using anti-IRS1 **(A)**, anti-IRS1-P^S307^ **(B)**, anti-IRS1-P^S612^ **(C)**, anti-PI3K **(D),** and anti-mTOR-P^S2448^ **(E)**. Values represent n=3-6, mean +/- SEM. Data were normalized to the empty vector-transfected control. Groups were compared using One-way ANOVA and Tukey’s *post hoc* test, p < 0.05 (*), p < 0.01 (**), p < 0.001 (***).

Hyperthyroidism is often accompanied by insulin resistance (33). In addition, *SMTNL1^-/-^* mice have impaired glucose tolerance associated with pronounced insulin resistance (24). Therefore, the effects of SMTNL1 on crucial members of insulin signaling were investigated. Although IRS1 expression did not change under any conditions (**Figure 6A**), IRS1 phosphorylation at Ser307 (**Figure 6B**) and Ser612 (**Figure 6C**) was increased following T3 treatment by 1.6-fold and 1.8-fold, respectively. SMTNL1 overexpression alone and in combination with T3 treatment reduced IRS1-P^S307^ to 48% and 73%, respectively. IRS1-P^S612^ decreased by 53% in response to combined treatment compared with IRS1-P^S612^ in the T3-treated cells (**Figures 6B, C**). Another important member of the insulin signaling pathway is PI3K. T3 treatment significantly decreased expression of the p85 subunit of PI3K but the other treatments did not significantly change p85 expression (**Figure 6D**). T3 treatment caused a negligible decrease in the phosphorylation of mTOR-P^S2448^ and overexpression of SMTNL1 had no effects. However, mTOR-P^S2448^ was increased significantly after combined treatment with T3 and SMTNL1 overexpression compared to the T3-treated cells (**Figure 6E**). Besides that, expression of mTOR remained unchanged under all conditions (**Figure S5A**). Collectively, these data provide evidence that T3 excess triggers the development of insulin resistance while overexpression of SMTNL1 can moderate these effects.

### Regulation of Ser/Thr Kinases Involved in Insulin Signaling by SMTNL1 Under Supraphysiological T3 Exposure in C2C12 Myotubes

**Figure 7:**
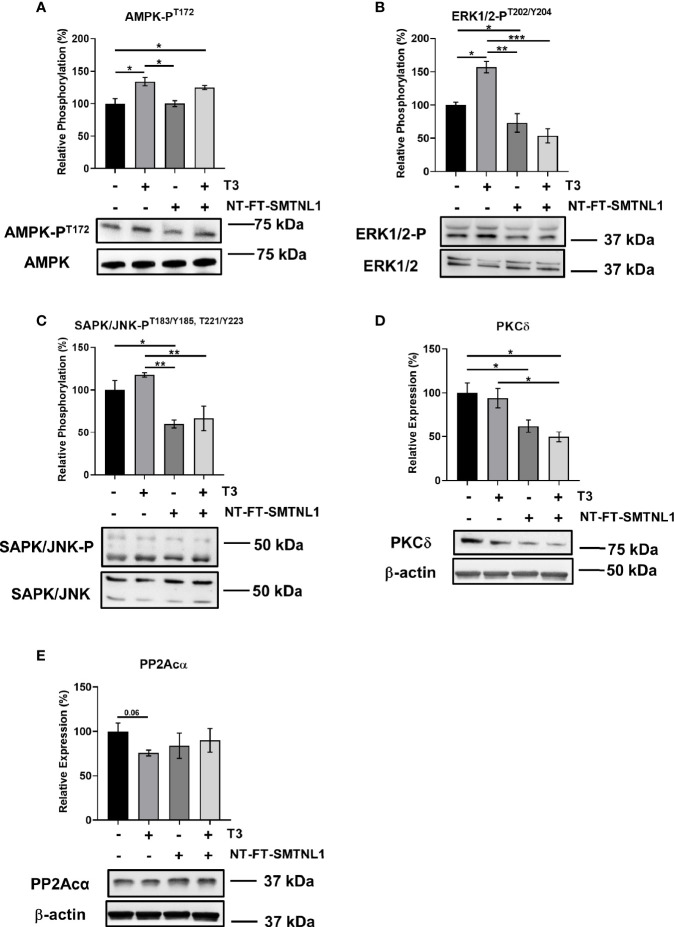
T3 excess and SMTNL1 overexpression regulate the phosphorylation and expression of various Ser/Thr kinases and phosphatases. **(A–E)** Myoblasts were transfected with either empty vector or NT-FT-SMTNL1 and were differentiated in parallel with a 72-hour T3 treatment from Day 4 onwards. Whole-cell lysates were separated by SDS-PAGE followed by Western blot analysis using anti-AMPK-P^T172^ **(A)**, anti- ERK1/2-P^T202/Y204^ **(B)**, anti-SAPK/JNK-P^T183/Y185,T221/Y223^ **(C)**, anti-PKCδ **(D),** and anti-PP2Acα **(E)** antibodies. Values represent n=4-6, mean +/- SEM. Data were normalized to the empty vector-transfected control. Groups were compared using One-way ANOVA and Tukey’s post hoc test, p < 0.05 (*), p < 0.01 (**), p < 0.001 (***).

The involvement of SMTNL1 in hyperthyroidism-induced posttranslational alterations was determined using Western blot analyses of NT-FT-SMTNL1-transfected and/or T3-treated myotubes. The expression of AMPK, ERK1/2 and SAPK/JNK did not change significantly (**Figure S5B–D**). AMPK-P^T172^ significantly increased by approximately 30% in response to T3 treatment alone or in combination with SMTNL1 overexpression (**Figure 7A**). In parallel, ERK1/2^T202/Y204^ phosphorylation increased by 57% in response to T3 treatment, while SMTNL1 overexpression induced a 27% decrease compared with phosphorylation in the non-treated control (**Figure 7B**). Although SAPK/JNK-P^T183/Y185,T221/Y223^ was elevated after T3 treatment, SMTNL1 overexpression alone reduced SAPK/JNK-P^T183/Y185,T221/Y223^ by 40%. Moreover, SMTNL1 overexpression was able to prevent the increased SAPK/JNK-P^T183/Y185,T221/Y223^ induced by T3 (**Figure 7C**). PKCδ expression did not change in response to T3 treatment, but decreased by 38% and 50% in response to SMTNL1 overexpression in the absence or presence of T3, respectively (**Figure 7D**). Finally, PP2Acα expression showed a marginally significant decrease in response to prolonged T3 treatment; SMTNL1 overexpression alone or in combination with T3 did not change PP2Acα expression (**Figure 7E**). Since IRS1 is a target of the previously mentioned Ser/Thr kinases, these data explain the elevated Ser phosphorylation levels of IRS1 in response to excess T3 and confirm the insulin-sensitizing effect of SMTNL1.

### SMTNL1 Overexpression Induces GLUT4 and Decreases Hexokinase II Expression in the Presence of Excess T3 in C2C12 Myotubes

**Figure 8:**
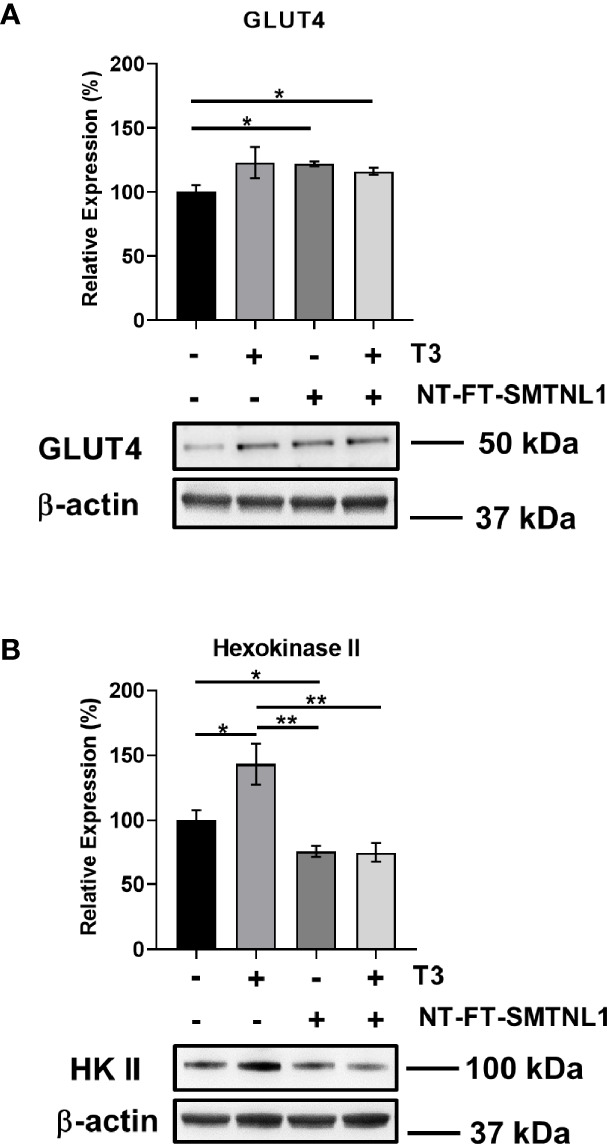
T3 treatment and SMTNL1 overexpression affect the expression of crucial members of glucose metabolism. Myoblasts were transfected with empty vector or NT-FT-SMTNL1 and were differentiated with a 72-hour T3 treatment starting from Day 4. Proteins from whole-cell lysates were analyzed by Western blot using anti-GLUT4 **(A)** and anti-hexokinase II **(B)** antibodies. Values represent n=3-4, mean +/- SEM. Data were normalized to the empty vector-transfected control. Groups were compared using One-way ANOVA and Tukey’s *post hoc* test, p < 0.05 (*), p < 0.01 (**).

Hyperthyroidism is a hypermetabolic state associated with elevated glucose utilization (33). Furthermore, deletion of *SMTNL1* causes impaired glucose tolerance *via* decreased GLUT4 levels (24). Therefore, we investigated the effects of SMTNL1 on important contributors to glucose metabolism. GLUT4 expression was markedly elevated under each condition (**Figure 8A**). In addition, T3 treatment significantly enhanced hexokinase II (HK II) expression by 43%. In contrast, HK II protein levels fell by 25% in response to SMTNL1 overexpression alone or in combination with T3 compared with the expression in the non-treated control (**Figure 8B**) suggesting that SMTNL1 plays a role in the regulation of glycolysis.

### Antagonistic Effects of SMTNL1 Overexpression on the Acidification Rate Upon T3 Overload in C2C12 Myoblasts

**Figure 9:**
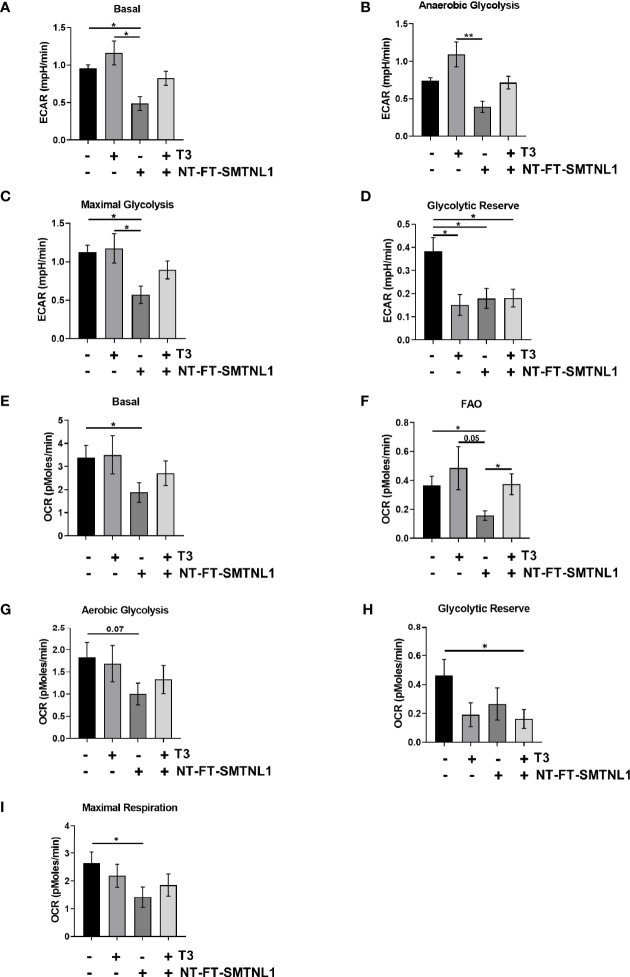
Measurement of Extracellular Acidification Rate (ECAR) and Oxygen Consumption Rate (OCR) by SeaHorse Analysis of C2C12 Myoblasts. **(A–I)** Following a 72-hour T3 treatment of empty vector or NT-FT-SMTNL1-transfected myoblasts, XF assay media supplemented with glucose was added to the cells. Myoblasts and the previously hydrated XF sensor cartridge loaded with inhibitors, etomoxir (50 µM), oligomycin (2 µM), FCCP (4 µM) and antimycin (10 µM) + 2-deoxy-D-glucose (100 mM), were incubated in a non-CO_2_ incubator before the assay. The rate of proton concentration increase **(A–D)** and the rate of oxygen concentration decrease **(E–I)** were measured. Values represent 4 groups with 23 technical replicates/group, n=2, mean +/- SEM. Data were normalized to protein content. Groups were compared using One-way ANOVA and Tukey’s post hoc test, p < 0.05 (*), p < 0.01 (**).

The rate of lactate production and mitochondrial respiration was investigated using the rate of increase in proton concentration by measuring the extracellular acidification rate (ECAR) or the rate of decrease in oxygen concentration (OCR). Three key parameters of cell energy metabolism were examined: baseline phenotype, stressed phenotype, and metabolic potential (**Figure S6A, B**). ECAR was markedly increased in response to T3 treatment, while SMTNL1 overexpression alone or in combination with T3 strongly decreased ECAR (**Figure 9A**). Similarly, anaerobic glycolysis was increased in response to excess T3, but decreased by half in response to SMTNL1 overexpression compared with the non-treated control. Moreover, SMTNL1 overexpression was able to prevent anaerobic glycolysis from increasing in response to excess T3 (**Figure 9B**). Excess T3 did not affect maximal glycolysis but SMTNL1 overexpression alone or in combination with T3 reduced maximal glycolysis (**Figure 9C**). Interestingly, the glycolytic reserve was diminished under each condition (**Figure 9D**). T3 treatment did not affect basal OCR, while SMTNL1 overexpression significantly reduced basal OCR (**Figure 9E**). The rate of mitochondrial respiration did not change in response to T3 treatment; however, SMTNL1 overexpression caused a 46% decrease in the rate of mitochondrial respiration (**Figures 9G–I**). Fatty acids can also be metabolized to pyruvate, acetyl CoA, and other intermediates for ATP production. Our results show that fatty acid oxidation (FAO) increased in response to excess T3, while SMTNL1 overexpression significantly reduced fatty acid oxidation by 58% (**Figure 9F**). Taken together, these data suggest that T3 overload strongly increases the rate of acidification (ECAR), while SMTNL1 overexpression antagonizes the T3 effects preventing glycolytic metabolism.

## Discussion

Our study is the first to compare global SKM gene expression in hyperthyroid and euthyroid patients. This screen revealed the role of a major regulator, SMTNL1, in thyroid homeostasis in SKM. In this study, we characterize the role of SMTNL1 in the molecular mechanism of hyperthyroidism in human skeletal muscle and the effect of supraphysiological T3 exposure on differentiated C2C12 muscle cells.

A Gene Ontology functional enrichment analysis of microarray data comparing SKM from euthyroid and hyperthyroid patients showed that hyperthyroidism negatively associated with mTOR, AMPK, PI3K/Akt, p70S6K and MAPK signaling (**Figure S2**). Moreover, hyperthyroidism-related differentially expressed genes were significantly involved in metabolic and energy production diseases, cellular and tissue development disorders, and defects in gene expression regulation. These results are in line with the results of Clément et al., who conducted a microarray analysis of five healthy men after a 14-day treatment with THs (34). However, our data are the first to describe changes in patients with long-standing hyperthyroid disease (**Figures 1** and **2**).

SMTNL1, as a transcriptional cofactor, regulates primarily progesterone and androgen receptors (35). No protein-protein interactions between TRα/β and SMTNL1 were detected in the presence of T3 (**Figure S4A**); thus, we ruled out the possibility of SMTNL1 being a member of the coregulatory complex of TH receptors. Data on human euthyroid and hyperthyroid samples demonstrated that elevated T3 levels drastically decreased SMTNL1 expression (**Figure 3A**). In addition, SMTNL1 expression was inversely proportional to T3 during the course of C2C12 differentiation (**Figure 4C**) but T3 caused a significant decrease in SMTNL1 expression only in differentiated myotubes (**Figure 4C** and **Figure S3C**) suggesting a mechanism related to developed SKM. Moreover, the activation of PKA resulted in a significant decrease in TRα expression, especially in cells overexpressing SMTNL1 (**Figure S4B**), suggesting that PKA-related SMTNL1 phosphorylation induced the translocation of SMTNL1 to the nucleus as it was described before (23) and triggered its transcriptional activity towards TRα expression. One possible explanation is that T3 acts through a non-genomic activation of kinases, which phosphorylate and activate transcription factors resulting in transcriptional regulation (15). The promoter of SMTNL1 contains binding sites for GR, AP-1, RXR, MyoD, and ER transcription factors (36). Thus, another possible explanation is that TR is tethered to DNA by other proteins, as the DNA-binding capacity of TR is dispensable and T3-bound TRs can modulate the function of AP-1 complexes without binding to DNA (37, 38). TRα downregulation by SMTNL1 overexpression and T3 treatment suggests that TRβ may be a potential transcription factor for SMTNL1. Lastly, MyoD could interact with TRs affecting the expression of SMTNL1 through an interaction as an extensive “regulatory lattice” through which different pathways communicate and influence one another (38).

The role of SMTNL1 in SKM homeostasis and development is supported by several lines of evidence. First, adult skeletal muscle undergoes interconversion between types I, IIa, IIx and IIb fibers in response to physiological and pathological signals (39). In the absence of SMTNL1, which is expressed mainly in type IIa fibers, SKM transforms into type IIx/IIb fibers with a glycolytic phenotype in humans and rodents, leading to a drastic change in global metabolic properties (24). Hyperthyroidism also initiates a shift in expression to the next faster MyHC isoform, that is, MyHC I switches to IIa, IIa to IIx, or IIx to IIb, depending on the initial fiber type and this isoform switch is regulated by many factors including T3 (40, 41). We observed a significant decrease in MyHC IIa/IIx fiber markers in hyperthyroid human SKM (**Figure 3B**), while MyHCIIb were not investigated due to its absence in human SKM (42). MyHC I changes were not significant, probably due to the very low of type I fibers under normal conditions (9%) compared to type IIa (24.2%), IIx fibers (20.8%), and type IIb (44.2%) (43) in sternohyoid muscle. Our data show that during C2C12 differentiation, T3 increased desmin and MyHC expression, indicating a more prominent myotube formation and accelerated muscle cell differentiation. The expression of SMTNL1 increased parallel to SKM markers under physiological conditions (**Figures 4A–C**). These results are in line with previous data showing that type I fibers decrease and type IIa markers gradually increase during C2C12 cell differentiation (40).

Second, SMTNL1 also regulates normal muscle homeostasis and development in SKM by regulating T3 signaling elements. A drastic decrease in TRα expression was observed when SMTNL1 was overexpressed under physiological and supraphysiological conditions (**Figure 5C**). These results are in agreement with a microarray analysis of SMTNL1 KO murine SKM that showed a significant elevation of nuclear receptor families, such as TRs; the ratio of TRα/TRβ expression decreased in SKM in response to hyperthyroidism (3). However, SMTNL1 affected neither the local control of T4 and T3 uptake by MCT8 nor T4 and T3 activation by DIO2 in SKM. These results suggest that SMTNL1 plays a regulatory role in TH signaling during SKM differentiation and development through the regulation of gene expression.

The capacity of THs to alter muscle phenotype is obvious for muscle performance and development in hyperthyroidism, but the phenotypic changes also affect whole-body glucose homeostasis. Patients with hyperthyroidism have elevated endogenous glucose production and often have disrupted intermediary metabolism associated with insulin resistance (44, 45). Physiological T3 levels enhance insulin signaling indirectly through insulin-induced activation of IRS1 and PI3K without affecting insulin receptor (IR) protein levels or phosphorylation (46). SMTNL1 regulates the gene expression of insulin signaling elements and promotes glycolysis and insulin sensitivity (24). Herein, we also demonstrated the impact of SMTNL1 on the metabolic properties of SKM. Current evidence suggests that excessive Ser phosphorylation of IR or downstream signaling molecules play a pivotal role in the pathogenesis of insulin resistance in SKM. Phosphorylation of IRS1 at Ser307 and Ser612 prevents interactions between IRS1, IRβ, and PI3K, leading to defective insulin signaling (47). Supraphysiological levels of T3 elevate the phosphorylation of IRS1 at Ser307 and Ser612 residues and SMTNL1 overexpression attenuates Ser phosphorylation of IRS1, suggesting insulin-sensitizing effects of SMTNL1 (**Figures 6B, C**). In hyperthyroid conditions, the downregulation of SMTNL1 resulted in an increase in JNK activity and consequently IRS1^S307^ phosphorylation. As JNK expression was unchanged, changes in IRS1 Ser phosphorylation are likely due to altered expression of Dual Specific Phosphatase-9 (DUSP9). DUSP9 is positively regulated by SMTNL1 (24) and dephosphorylates and inactivates JNK (48). In prolonged T3 exposure, inhibition of SMTNL1 also caused an increase in PKCδ expression, another IRS-kinase (**Figure 7D**). PKCδ is associated with increased IRS1^S307^ (49) and ERK1/2 activation (50). ERK1/2 is also activated by elevated T3 exposure (**Figure 7B**) *via* a non-genomic effect and its activity is responsible for IRS1^S612^ phosphorylation (51).

Hyperthyroidism is a hypermetabolic state characterized by increased demand for glucose, which is provided by increased glucose transport and phosphorylation and lactate formation (19). Thyrotoxic subjects show increased glucose turnover with elevated peripheral glucose transport and utilization (20). SKM of hyperthyroid rats exhibited an increase in the number and translocation of GLUT4 to the sarcolemma in response to insulin (52, 53). In our model, prolonged T3 exposure enhanced the expression of GLUT4 suggesting its genomic action on glucose transport.SMTNL1 overexpression also elevate GLUT4 level. In addition, deletion of *SMTNL1* is known to reduce GLUT4 protein expression in SKM (24). Here, we show that SMTNL1 elicits insulin-sensitizing effects by markedly increasing GLUT4 expression and reducing Ser phosphorylation of IRS1 ameliorating the malfunction of insulin signaling (**Figures 5** and **7**). In this manner, SMTNL1 may be able to restore the normal responsiveness of glucose transport to insulin.

As insulin-stimulated rates of glycogen synthesis are reduced in muscle during hyperthyroidism, glucose residues are utilized by glycolysis and converted to glucose-6-phosphate catalyzed by hexokinases (HKs) (54). Both the expression and activity of HK II, the most abundant isoform in muscle, are increased in hyperthyroid muscle leading to enhanced glucose phosphorylation (55–57). HK II levels were elevated in hyperthyroid conditions and SMTNL1 overexpression attenuated this effect (**Figure 8B**). This result is supported by previous results in SMTNL1 KO mice showing an increase of HK II in SKM (24).

Hyperthyroidism also increases the rates of lactate formation and glucose oxidation in SKM; however, lactate formation is preferentially increased over glucose oxidation (58). The increase in acidification may be due to the enhanced rate of glycogenolysis observed in hyperthyroidism (19). Our data are supported by cellular bioenergetics measurements on DIO2-overexpressing C2C12 cells showing that increased intracellular T3 level caused a shift to glycolysis and increased ECAR (59). Similarly, deletion of *SMTNL1* also leads to elevated glycogenolysis in mice (24). Our data are in line with these findings; T3 overload strongly induced anaerobic glycolysis, which was accompanied by a fall in glycolytic reserve. T3 overload did not affect maximum glycolysis compared to the control. Interestingly, SMTNL1 overexpression not only prevented this increase in anaerobic glycolysis but also caused a massive drop in basal and maximal glycolysis (**Figure 9**).

Another regulator of metabolism through the regulation of mitochondrial biogenesis is AMP-activated protein kinase (AMPK), which is increased in SKM of hyperthyroid rats (28). The activation of AMPK evokes important downstream effects on non-genomic T3 signaling in the experimental hyperthyroid model of SKM (**Figure 7A**), resulting in chronic activation of AMPK and increased energy demand. In addition, PP2A expression is decreased (**Figure 7E**), leading to persistent phosphorylation of AMPK (60). Moreover, basal oxygen consumption and fatty acid oxidation were elevated in response to elevated T3. Of note, SMTNL1 did not affect AMPK activity, nor did SMTNL1 antagonize the T3 effects on AMPK. Based on these data, SMTNL1 appears to have not just a T3-antagonistic effect but also a role in metabolic regulation mainly through the control of glycolysis under hyperthyroid conditions.

**Figure 10:**
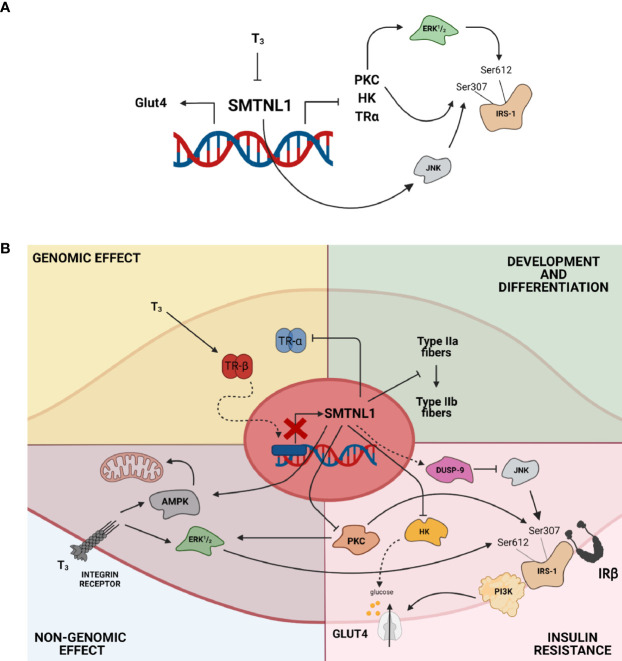
SMTNL1 maintains the homeostasis of SKM in hyperthyroidism. **(A, B)** SMTNL1 potentially prevents hyperthyroidism-induced changes in SKM *via* the following mechanisms: 1) SMTNL1 compensates for the glycolytic phenotype shift of SKM triggered by pathological T3 exposure; 2) SMTNL1 induces insulin sensitivity through an insulin-independent heterologous pathway by inhibiting the activity of JNK; 3) SMTNL1 hampers the non-genomic effect of T3 by decreasing the activity of ERK1/2 through PKCδ; 4) SMTNL1 regulates glucose phosphorylation and balances glycolysis and glycogen synthesis by the regulation of HK II; and 5) SMTNL1 selectively inhibits TRα expression, which is a key target of insulin-dependent signaling governed by T3.

SMTNL1 modulation holds great promise for treating hyperthyroidism for the following reasons: 1) SMTNL1 regulates muscle cell differentiation and compensates for the glycolytic shift in SKM triggered by pathological T3 exposure; 2) SMTNL1 induces insulin sensitivity through an insulin-independent heterologous pathway by inhibiting JNK activity; 3) SMTNL1 hampers the non-genomic effects of T3 by decreasing the activity of ERK1/2 through PKCδ; 4) SMTNL1 regulates glucose phosphorylation and balances glycolysis and glycogen synthesis *via* the regulation of HK II; and 5) SMTNL1 selectively inhibits TRα expression, which is a key target of insulin-dependent signaling governed by T3. The overexpression of SMTNL1 or an SMTNL1-mimicking membrane-permeable peptide might counteract the effects of hyperthyroidism selectively in SKM, promoting insulin-sensitizing effects and maintaining the homeostasis of SKM (**Figure 10**).

## Data Availability Statement

The datasets presented in this study can be found in online repositories. The names of the repository/repositories and accession number(s) can be found in the article.

## Ethics Statement

The studies involving human participants were reviewed and approved by Regional and Institutional Ethics Committee of the University of Debrecen and were in accordance with guidelines of the European Union Council and Hungarian regulations under license number DEOEC RKEB/IKEB 3517-2011. The patients/participants provided their written informed consent to participate in this study.

## Author Contributions

BL and PF: conceptualization. EM and BL: data curation. EM and IK: visualization. EM, DH, FG, and IT: methodology. FG: tissue collection. EM: prepared figures and analyzed data. PF and BL: funding acquisition. EM, PF, KU, and BL wrote and reviewed the manuscript. All authors contributed to the article and approved the submitted version.

## Funding

This work was supported by grants from the National Research, Development and Innovation Office (FK125043), the University of Debrecen, Faculty of Medicine Research Fund (1G3DBKJ0BFTK 247) for BL, from the EU co-financed by the European Regional Development Fund under project EFOP-3.6.2-16-2017-00006 and from the EFOP-3.6.3-VEKOP-16-2017-00009 project co-financed by EU and the European Social Fund. BL and PF MECENATURA from the University of Debrecen. 

## Conflict of Interest

The authors declare that the research was conducted in the absence of any commercial or financial relationships that could be construed as a potential conflict of interest.

## Publisher’s Note

All claims expressed in this article are solely those of the authors and do not necessarily represent those of their affiliated organizations, or those of the publisher, the editors and the reviewers. Any product that may be evaluated in this article, or claim that may be made by its manufacturer, is not guaranteed or endorsed by the publisher.

## Glossary

**Table:**

2-DG	2-deoxy-D-glucose
8BrcAMP	8-Bromoadenosine 3’,5’-cyclic monophosphate
AMPK	AMP-activated protein kinase
DIO2	iodothyronine deiodinase type 2
DMEM	Dulbecco’s Modified Eagle’s Medium
DUSP	dual specific phosphatase-9
ECAR	extracellular acidification rate
ECL	enhanced chemilumineswfi 2cence
ERK1/2	extracellular signal-regulated kinase
FAO	fatty acid oxidation
FBS	fetal bovine serum
FCCP	carbonyl cyanide-p-trifluoromethoxyphenylhydrazone
GLUT4	glucose transport molecule 4
GR	glucocortiocoid receptor
HK II	hexokinase II
HRP	horseradish peroxidase
HS	horse serum
IR	insulin receptor
IRS1	insulin receptor substrate 1
MCT8	monocarboxylate transporter 8
mTOR	mammalian target of rapamycin
MyHC	myosin heavy chain
MyoD1	myogenic differentiation 1
OCR	oxygen consumption rate
PI3K	phosphatidylinositol 3-kinase
PKA	protein kinase A
PKCδ	protein kinase C delta
PP2Aα	protein phosphatase 2A alpha
RIPA	radioimmunoprecipitation assay buffer
RXR	retinoid X receptor
SAPK/JNK	stress-activated protein kinase/Jun N-terminal kinase
SKM	skeletal muscle
SMTNL1	smoothelin-like protein 1
T3	3,3′,5-Triiodo-L-thyronine
T4	thyroxine
TH	thyroid hormone
TR	thyroid receptor
TRE	thyroid-responsive element
