# Supplementary material for: Smoothelin-Like Protein 1 Regulates Development and Metabolic Transformation of Skeletal Muscle in Hyperthyroidism
Source: Front Endocrinol (Lausanne). 2021 Oct 5;12:751488. doi: 10.3389/fendo.2021.751488 (PMC8524136; doi:10.3389/fendo.2021.751488)
Supplement: Supplementary file 1 [file DataSheet_1.docx]

**SUPPLEMENTARY MATERIALS**

**Smoothelin-like protein 1 Regulates Development and Metabolic Transformation of Skeletal Muscle in Hyperthyroidism**

Evelin Major^1^, Ferenc Győry^2^, Dániel Horváth^1^, Ilka Keller^1^, István Tamás^1^, Karen Uray^1^, Péter Fülöp^3^, Beáta Lontay^1*^

^1^Department of Medical Chemistry, Faculty of Medicine, University of Debrecen

^2^Department of Surgery, Clinical Centre, University of Debrecen

^3^Department of Internal Medicine, Clinic of Internal Medicine, Clinical Centre, University of Debrecen

***Correspondence to:**

Dr. Beáta Lontay

Department of Medical Chemistry, Faculty of Medicine

University of Debrecen,

H-4032 Debrecen, Egyetem tér 1. Hungary

Phone: +36-52-412345

e-mail: lontay@med.unideb.hu

**Running title:** Smoothelin-like Protein 1 in Hyperthyroidism

Number of characters: (with space, includes all sections).

**SUPPLEMENTARY MATERIALS AND METHODS**

**
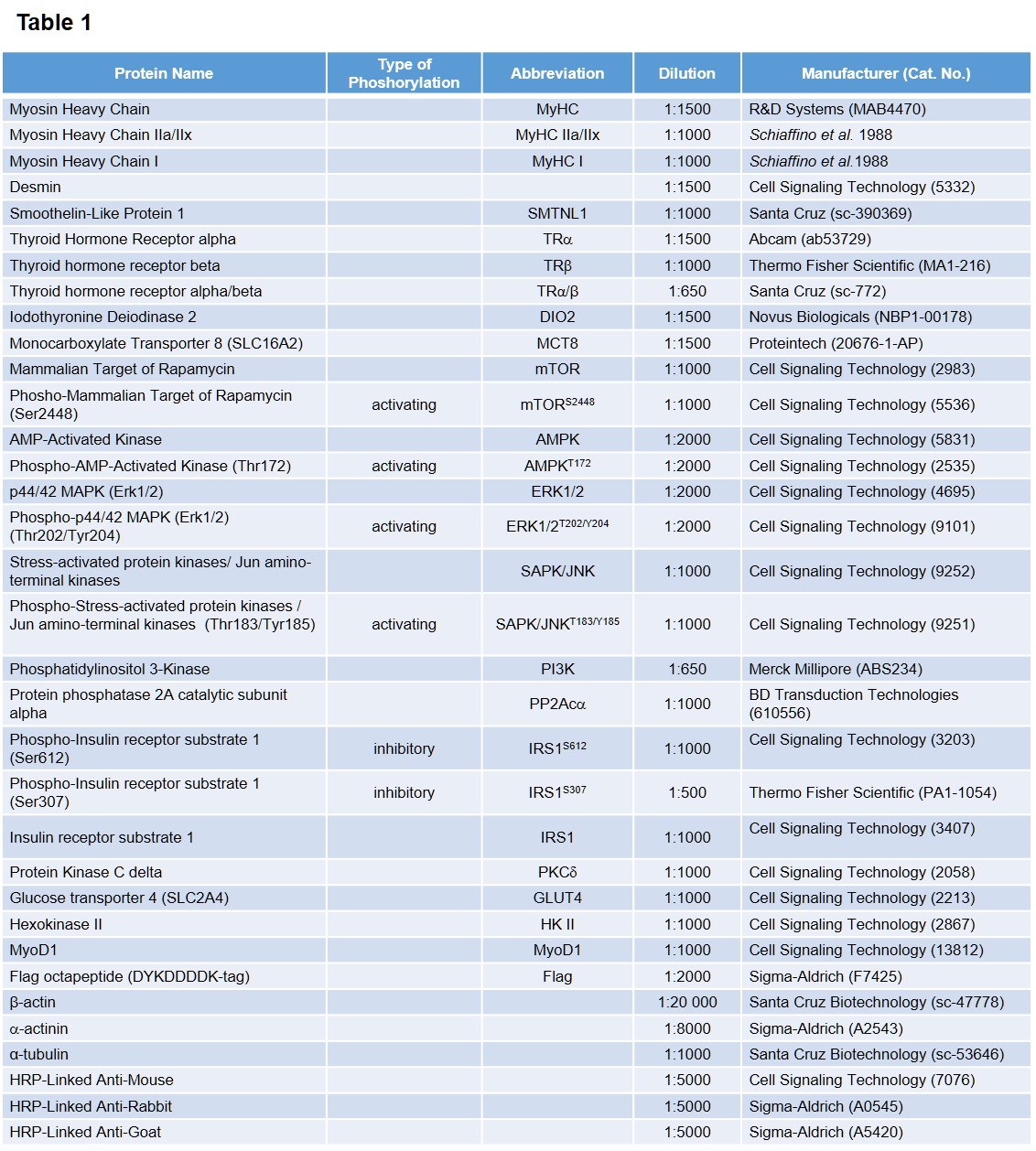
**

**Transient Transfection of Myoblasts with Empty Vector or NT-FT-SMTNL1.** 2 µg of plasmid DNA (empty vector: pEZ-M13 and N-terminal Flag-tagged SMTNL1 vector: pM13-NT-FT-SMTNL1) (GeneCopoeia Inc., Rockville, MD, USA) per well was added to serum-free DMEM/transfection reagent mixture and incubated at RT for 15 minutes. Then, the entire volume of the transfection mixture was added drop-wise to the cells in complete growth medium. Finally, cells were seeded at a density of 300 000 cells per well, unless otherwise indicated, in collagen-coated tissue culture plates or dishes and incubated at 37 °C overnight (O/N).

**AlamarBlue Cell Viability/Cytotoxicity Assay.** Myoblasts were transfected with either empty vector or NT-FT-SMTNL1 and plated at a density of 20 000 cells per well in collagen-coated 96-well plates. Following 6 days of differentiation combined with a 72-hour T3 treatment of cells in differentiation medium, alamarBlue reagent was added to each well at a final concentration of 20 µM and plates were incubated for 4 hours at 37 °C. Fluorescence was measured at 530/590 nm with a Spark multimode microplate reader (Tecan Group Ltd., Mannedorf, Switzerland). The intensity of fluorescence is proportional to the number of living cells.

**Immunoprecipitation of NT-FT-SMTNL1.** Anti-Flag M2 Affinity Gel was blocked with 5% BSA/1x TBS supplemented with 10x protease inhibitor cocktail at 4 °C O/N on a roller shaker. NT-FT-SMTNL1-transfected C2C12 cell lysates, treated with 10 nM T3 for 24 hours or 10 µM 8BrcAMP for 30 minutes, were incubated with Protein-A Sepharose for 1 hour. Precleared cell lysates (200 µl) were added to the blocked resin and samples were agitated for 2 hours. Elution of recombinant proteins was carried out by boiling the samples with 1x SDS buffer for 5 minutes. Samples were analyzed by Western blot as described in the *Western Blot Analysis* section.

**8BrcAMP Treatment of NT-FT-SMTNL1-transfected C2C12 Myotubes.** Differentiated myotubes overexpressing NT-FT-SMTNL1 were treated with a membrane-permeable PKA agonist, 8BrcAMP, at a final concentration of 10 µM for 24 hours. Then, cells were harvested, lysed, and subjected to Western blot analysis.

**Membrane Stripping with Heat and Detergent.** Western blot membranes were washed 3 times for 5 minutes with 1x TBST and incubated in Stripping buffer [0.5 M Tris-HCl pH 6.8; 10% SDS, β-mercaptoethanol] at 50 °C for 30 minutes in a Thermo-Shaker (Biosan Laboratories Inc., Warren, MI, USA). Next, membranes were washed 6 times for 5 minutes with 1x TBST and reblocked with 5% BSA/TBST at RT for 1 hour. Incubation with primary antibodies followed by secondary antibodies and visualization of immunoreactions were carried out as described in the *Western Blot Analysis* section.

**Horse Radish Peroxidase (HRP) Inactivation.** This method, developed by Sennepin and coworkers [1], involves irreversible inhibition of HRP activity by its substrate, hydrogen peroxide (H_2_O_2_), allowing multiple reprobing of membranes without losing considerable amounts of blotted proteins. Membranes were washed 3 times for 5 minutes with 1x TBST and incubated with 1 ml of 30% (w/v) H_2_O_2_ solution at 37 °C for 15 minutes without shaking. Then membranes were washed 6 times for 5 minutes each with 1x TBST and re-blocked with 5% BSA/TBST at RT for 1 hour. Incubation with primary antibodies followed by secondary antibodies and visualization of immunoreactions were carried out as described in the *Western Blot Analysis* section.

**SUPPLEMENTARY FIGURE LEGENDS**

***Figure S1.*** *Statistical Data Analysis of Euthyroid and Hyperthyroid Patients* Data were analyzed using Excel for obtaining gender and age distribution of euthyroid and hyperthyroid patients (Table 1). Data were also analyzed using a Body Mass Index (BMI) calculator for obtaining the BMI of euthyroid and hyperthyroid patients based on their height, weight, age, and gender (Table 1).

***Figure S2.*** *Significantly Altered Signaling Pathways Revealed by Microarray Analysis* The most significantly altered signaling pathways and their regulated genes from microarray analysis of euthyroid and hyperthyroid SKM biopsies. Green indicates a decrease, while red indicates an increase in gene expression.

***Figure S3.*** *Effects of T3 Treatment and SMTNL1 Overexpression on the Viability of Differentiated C2C12 Cells* Myoblasts were transfected with empty vector or NT-FT-SMTNL1 and differentiated with a simultaneous 72-hour T3 treatment starting from Day 4. **(A)** SMTNL1 overexpression was confirmed by Western blot analysis using anti-Flag antibody specific for recombinant N-terminal Flag-tagged SMTNL1 protein. **(B)** 20 µM alamarBlue reagent was added and cells were incubated for 4 hours. Fluorescence was measured at 530/590 nm. **(C)** C2C12 cells were treated with 10 nM T3 for 72 hours from Day 4 of differentiation onwards. Whole-cell lysates were analyzed by Western blot using an anti-SMTNL1 antibody. Values represent n=3-4, mean +/- SEM. Data were normalized to the empty vector-transfected control. Groups were compared using One-way ANOVA followed by Tukey’s *post hoc* tests and unpaired two-tailed t-tests, ns = non-significant and p <0.01 (**), p <0.0001 (****).

***Figure S4.*** *The Relationship Between SMTNL1 and Thyroid Hormone Receptors* **(A)** Immunoprecipitation of Flag-SMTNL1 with TRα/β upon T3 or 8BrcAMP treatment. Samples were analyzed by Western blot using anti-Flag and anti-TRα/β antibodies. **(B)** Myoblasts were transfected with empty vector or NT-FT-SMTNL1 and differentiated for 6 days followed by a 24-hour treatment with 8BrcAMP. Samples were analyzed by Western blot using an anti-TRα antibody. Values represent n=3, mean +/- SEM. Data were normalized to the empty vector-transfected control. Groups were compared using One-way ANOVA and Tukey’s *post hoc* tests, p <0.05 (*), p <0.01 (**).

***Figure S5.*** *Expression Levels of Various Ser/Thr Kinases in T3-treated and/or NT-FT-SMTNL1-transfected C2C12 Myotubes* Myoblasts were transfected with either empty vector or NT-FT-SMTNL1 and differentiated for 6 days with a 72-hour T3 treatment starting from Day 4. Proteins from whole-cell lysates were analyzed by Western blot using anti-mTOR **(A)**, anti-AMPK **(B)**, anti-ERK1/2 **(C),** and anti-SAPK/JNK **(D)** antibodies. Values represent n = 5-6, mean +/- SEM. Data were normalized to the empty vector-transfected control. Groups were compared using One-way ANOVA and Tukey’s *post hoc* tests, ns = non-significant.

***Figure S6.*** *Schematic Representation of Cellular Bioenergetics* **(A-B)** Cells generate energy in the form of ATP through two major energy-producing metabolic pathways: glycolysis and oxidative phosphorylation. Glycolysis metabolizes glucose to pyruvate, which can be further reduced to lactate or oxidized via the TCA cycle and the mitochondrial electron transport chain (mETC) to generate ATP. Fatty acids and amino acids can also be broken down to pyruvate, acetyl CoA, and other intermediates for ATP production. The glucose analog, 2-deoxy-D-glucose, cannot be converted to glucose-6-phosphate. Therefore, 2-deoxy-D-glucose competitively inhibits glycolytic ATP production. Antimycin A blocks complex III of the mETC, while oligomycin inhibits complex V by blocking its proton channel. Carbonyl cyanide-p-trifluoromethoxyphenylhydrazone (FCCP) is an uncoupling agent that permeabilizes the inner mitochondrial membrane to protons, forcing the mitochondria to increase the flow of electrons (and thus oxygen consumption) to maintain the membrane potential. Etomoxir is a widely used small molecule that irreversibly inhibits carnitine palmitoyltransferase 1 (CPT1) located in the outer mitochondrial membrane through which it stops fatty acid oxidation. The rate of lactate production **(A)** and mitochondrial respiration **(B)** measured with the SeaHorse Extracellular Flux Analyzer are framed with red lines.

***Figure S7.*** *The Effect of T3 Treatment and SMTNL1 Overexpression on β-actin Expression* Myoblasts were transfected with either empty vector or NT-FT-SMTNL1 and differentiated for 6 days with a 72-hour T3 treatment starting from Day 4. Whole-cell lysates were analyzed by Western blot using β-actin antibody as a loading control. Values represent pixel densities from densitometric analysis, n=3, mean +/- SD. Groups were compared to the empty vector-transfected control using One-way ANOVA followed by Tukey’s *post hoc* test. P-values are shown above the bars.

**SUPPLEMENTARY FIGURES**

**
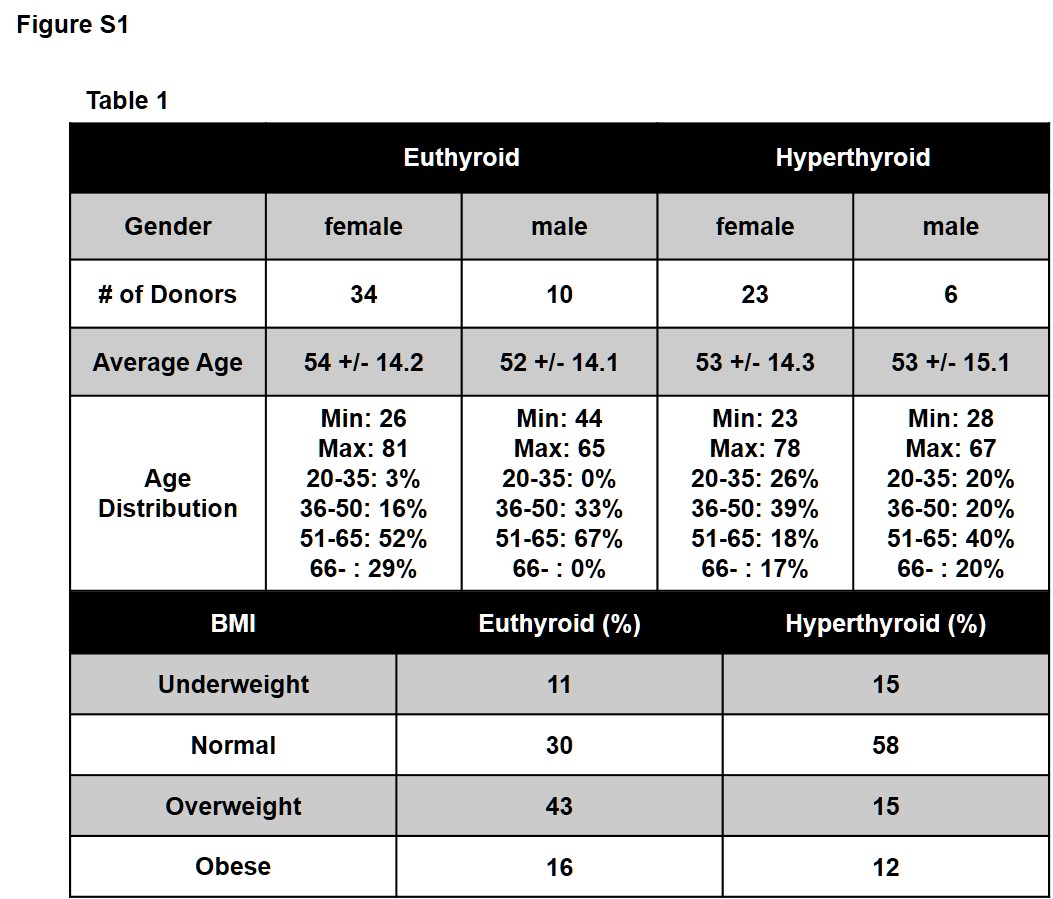
**

**
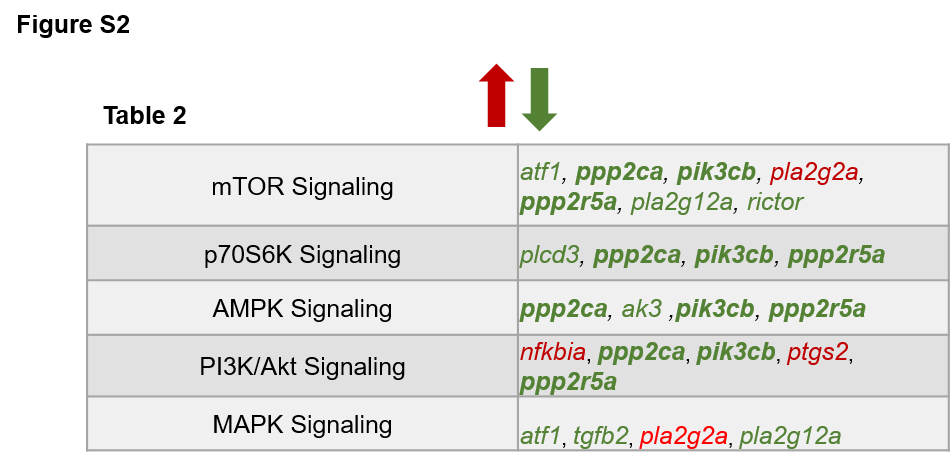
**

**
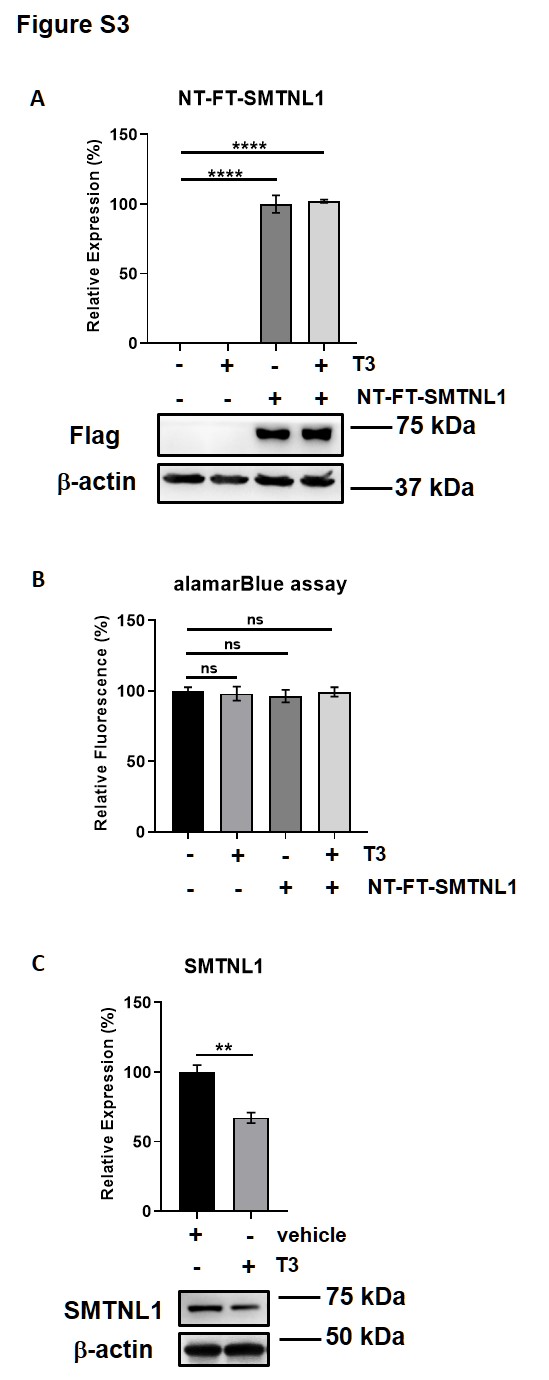
**

**
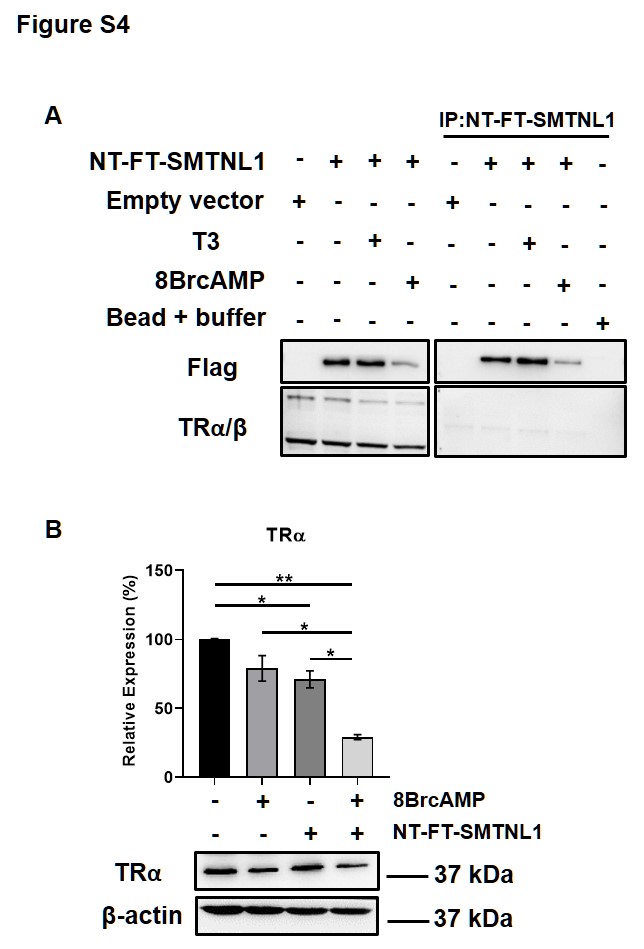
**

**
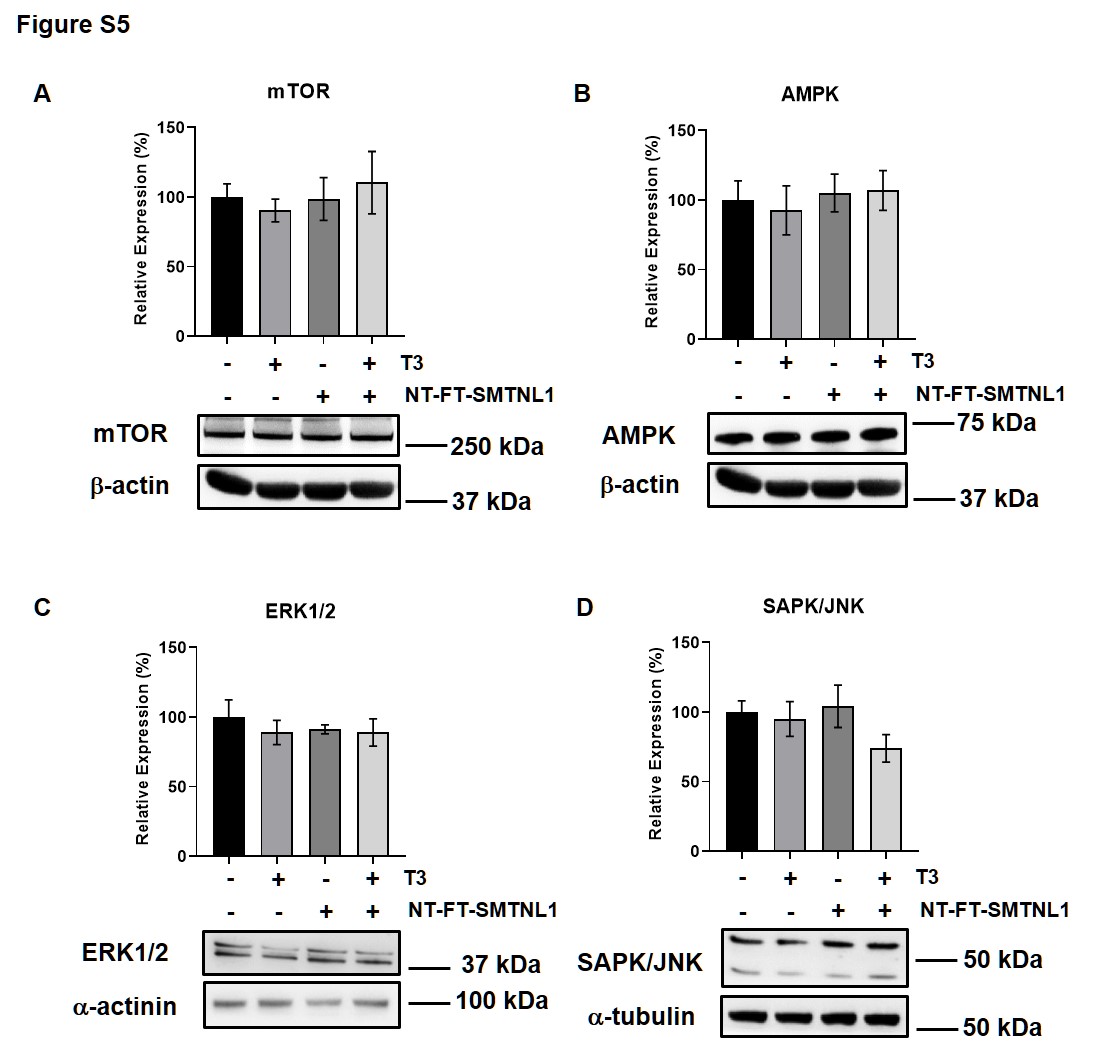
**

**
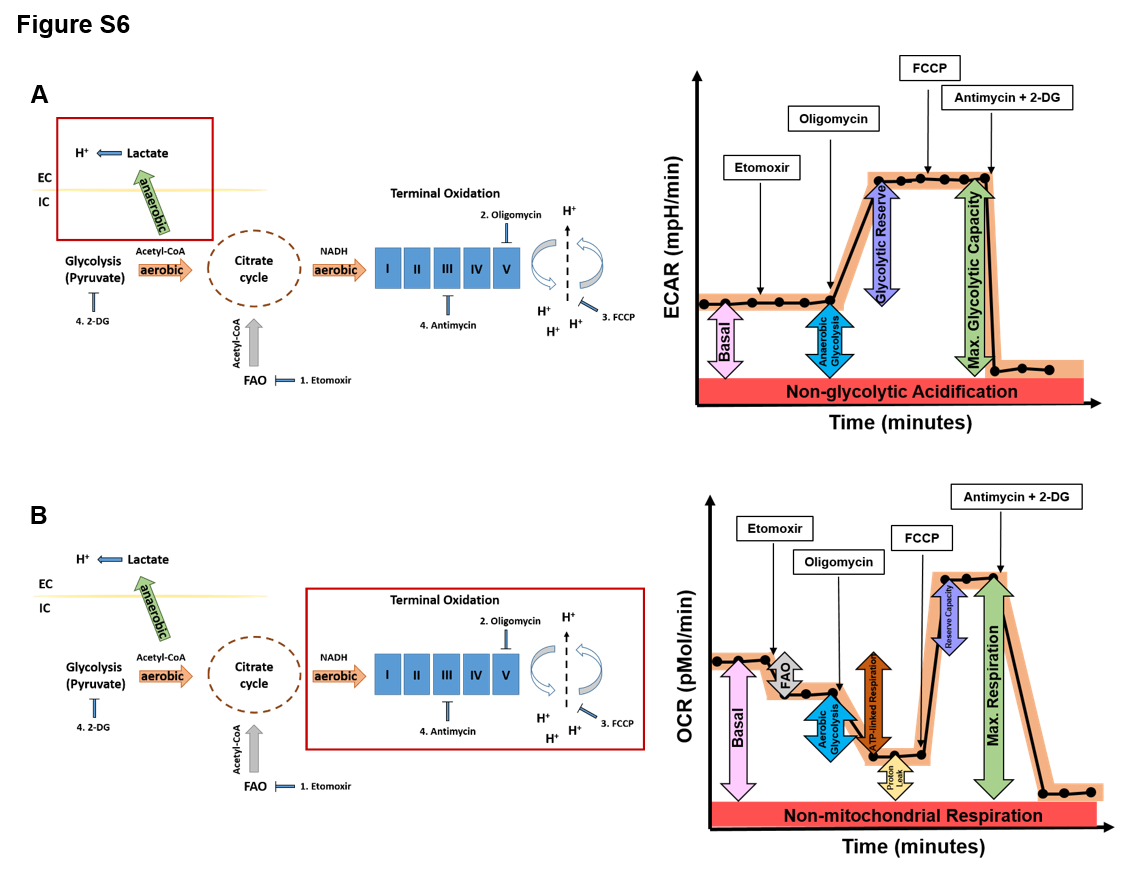
**

**
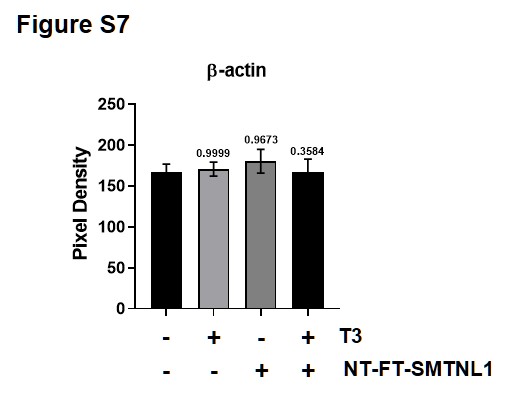
**

**SUPPLEMENTARY REFERENCES**

[1] A.D. Sennepin, S. Charpentier, T. Normand, C. Sarre, A. Legrand, L.M. Mollet, Multiple reprobing of Western blots after inactivation of peroxidase activity by its substrate, hydrogen peroxide, Anal Biochem, 393 (2009) 129-131.
